# Major congenital malformations and residential proximity to a regional industrial park including a national toxic waste site: An ecological study

**DOI:** 10.1186/1476-069X-5-8

**Published:** 2006-03-29

**Authors:** Yaakov Bentov, Ella Kordysh, Reli Hershkovitz, Ilana Belmaker, Marina Polyakov, Natasha Bilenko, Batia Sarov

**Affiliations:** 1 Department of Obstetrics & Gynecology, Soroka University Medical Center, POB 151 Beer-Sheva, Israel; 2 Department of Epidemiology and Health Services Evaluation, Faculty of Health Sciences, Ben-Gurion University of the Negev. POB 653, Beer-Sheva, Israel; 3 Ministry of Health, Southern Region, POB 10050, Beer-Sheva, Israel

## Abstract

**Background:**

Public concern about exposure to emissions from the regional industrial park (IP), including 17 chemical plants and the national industrial toxic waste site, initiated this study of the possible association between major congenital malformations (MCM) and residence near the IP in Israel's Southern District.

**Methods:**

The study was conducted during the period 1995–2000 and included 63,850 deliveries. Data on deliveries and MCM detected at births were obtained from the regional medical center, and stratified by ethnicity and type of locality. As exposure indicator we used distance categories (proximal and distant) and predominant wind direction from the IP. Distance stratification was based on the geographical distribution of the localities and complaints about the odor related to IP emissions. Based on these complaints, localities up to 20 km from the IP were considered proximal to the IP.

**Results:**

Average rates of MCM were 5.0% and 4.1% for Bedouin and Jewish newborns, respectively. The rate of MCM for Bedouin from proximal localities was significantly greater compared with distant localities (5.6% *vs. *4.8%; RR = 1.17 with 95% CI: 1.04–1.29). In the proximal Bedouin permanent localities, the MCM rate reached 8.2 %, which was significantly higher than in distant areas (RR = 1.63, 95% CI: 1.39–1.80). Significant risk increase of central nervous system MCM was found in these localities, compared to distant ones (RR = 2.27, 95% CI: 1.44–3.60). Among newborns from the traditional tribal settlements, proximity to the IP was associated with increased rates of the following MCM: 1) all combined, 2) those associated with chromosomal abnormalities, and 3) those defined as "others unclassified MCM." Comparison of autosomal recessive disease rates by proximity to the IP in Bedouin newborns indicates that the observed increased risk of MCM is not explained by consanguineous marriages. The rates of MCM in the Jewish population were similar among "exposed" and "unexposed" inhabitants.

**Conclusion:**

Residential proximity to the IP is associated with increased rates of MCM among Arab-Beduin but not in Jewish populations. These observations indicate the need for public health protection of a vulnerable society in transition, although the relative importance of chemical exposure and health care utilization requires further study.

## Background

The incidence of congenital malformations is 3–5% of all births and their etiology is often not identifiable [1]. The etiology of congenital malformations has been found to be genetic in 20–25% of these cases. The pathophysiology of the majority of birth defects is unknown. Nevertheless, many congenital malformations are presumed to be the consequence of a complex interaction between genetic predisposition and fetal environmental factors, so-called multifactorial inheritance.

The regional industrial park (IP) in the Beer-Sheva subdistrict of Southern Israel consists of 17 chemical facilities and has served as a major employer in this area for the last 25 years. Since 1976, two of the largest chemical plants in the country have been located here. Various products of the IP facilities include pesticides, combustion-inhibiting agents, bromic acid, HCl, hydrogen peroxide, raw material for drugs, metal recycling from metal waste, liquid nitrogen, oxygen, and argon, and raw material for the plastic industry (by recycling consumable plastic).

For the past 17 years, the IP has also included the national industrial hazardous waste disposal site. Every year, 35,000 tons of toxic wastes are transferred to the site. A few years ago, an incinerator for organic waste was erected, burning more than 20,000 tons of waste every year.

The list of emissions from industrial facilities and evaporation pools at the IP includes a variety of aliphatic, aromatic, and polycyclic hydrocarbons as well as several dozen inorganic agents (including heavy metals). Every 30 minutes the following air pollutants are monitored at the IP site: H_2_S, Cl, Br, HBr, and HCl. Every 24 hours, the concentrations of 61 organic chemicals (benzene and its halogenated, methylated, and ethylated derivates, benzaldehyde, hydroxy benzaldehyde, benzo nitrile, cyclohexene, dimethylamine, naphthalene, tetrahydronaphtalene, toluene, xylene, bromo xylene, acetone, terpinene, methane, ethane, and ethene halogenated compounds, carbon disulfide and tetrachloride, chloroform, sulfides of dimethyl, ethyl acetate, ethylene bromide, isopropanol, methylene chloride, methyl-ethyl-ketone, methyl-iso-butyl-ketone, methyl-phenyl-ketone, phosphorodithioic acid trimethyl ester, tetra-chloro-pyridine, acetic acid) are measured.

According to the periodic reports of the IP authorities to the Regional Ministry of Health, most of the measurements, conducted by the IP Department of Environment Protection, found low concentrations of pollutants at the IP site, several orders of magnitude below the limit allowed for occupational exposure Even under the assumption that proportions between occupational threshold level values (TLV) and measurement results reflect "safety coefficients" for populated places, one should also take into account the possible additive or synergistic health effects of the combination of several pollutants, even though each one of them does not exceed the allowed limit for the exposed population. Furthermore, although a large number of pollutants are measured, there is a possibility that other potentially toxic pollutants are being emitted without proper monitoring. Ambient air quality monitoring (of criteria pollutants) is performed only in Beer-Sheva, the sub district's capital city.

The region's semi-arid climate, characterized by frequent temperature inversions and small amount of annual precipitation (15 to 300 mm), reduce the air's self-clean-up. The reports of emissions from the IP include solvents, pesticides, heavy metals, dioxins, and CS_2_, which have been identified as potential human developmental toxicants in experiments on animals and studies of human exposure to high levels of chemicals in their occupational environment [2]. Exposure to chemical plant emissions has been considered to be related to increased rates of birth defects among residents of the surrounding areas [3-9]. Pesticides, one of the components of the IP emissions, are associated with elevated rates of congenital anomalies in newborns in communities with potential exposure to this air pollutant from agriculture, industry, and waste sites [10-14].

The health implication of the complex chemical mixtures originating from chemical waste sites and large chemical plants is being intensively studied by the Agency for Toxic Substances and Disease (ATSDR). A recently published study [15] describes an increased rate of MCM among ethnic minorities living in California near hazardous waste sites. The highest rate of birth defects among residents of the proximal neighborhoods was among American Indians/Alaska Natives. Another study conducted by the ATSDR [16] described an alteration of gene expression under the influence of a certain chemical mixture, as a possible mechanism for birth defects.

The multiplicity of the regional sources of hazardous chemical pollutants, including that derived from the emissions of 17 chemical plants and the national industrial toxic waste site, undoubtedly deserves special attention. This study was initiated by the Israel Ministry of Health, following complaints of residents of surrounding localities who blame the IP emissions for the odor nuisance and suspect that possible long- or short-term health disorders could be attributed to this exposure. The hypothesis of this study is that incidence rates of major congenital malformations (MCM) among newborns in the vicinity of the IP may be higher than in newborns from the remote areas.

## Methods

The completely ecological study was conducted during the period 1995–2000. The study population comprised of 63,850 newborns (including 511 stillborn) who were born during the period of the study in the Soroka University Medical Center, the only hospital in the Beer-Sheva subdistrict. This sample was stratified by ethnicity (Jews and Bedouins). The reasons for this stratification were significant differences in lifestyle, socio-economic level, and health indices, such as higher infant mortality and higher MCM rates observed in the country's Bedouin population [17,18].

Population subgroups were divided by type of locality; for the Jewish population: urban localities, including the city of Beer-Sheva, urban satellite localities of Beer-Sheva, and agricultural localities; for the Bedouin population: permanent localities and traditional tribal settlements.

Bedouin permanent localities are suburban settlements, erected by the state as part of an effort to stabilize the semi-nomadic Bedouin tribes. These settlements provide modern residence and municipal services. The traditional tribal settlements, on the other hand, are widespread spontaneous amassments. The inhabitants practice arid land agriculture and reside in tents or shacks with no running water or electricity. Preventive health care is provided by mobile health care units. Nevertheless, most Bedouin families own a vehicle in addition to the well-developed public transportation.

Medical care in Israel, including maternal prenatal care clinics, is provided in accessible community clinics at no or minimal cost to the patient. The Bedouin population is known to highly utilize the medical services.

**Figure 1:**
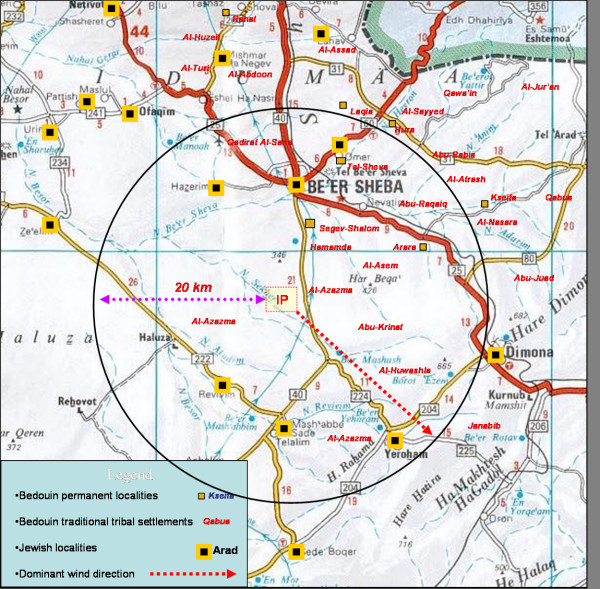
Distribution of major localities according to the 20 km boundary.

As an exposure indicator we used distance from the IP (Figure 1). The distance stratification was based on the geographical distribution of the localities and complaints about the odorous pollutants related to the IP emissions. Since odor nuisance is a serious problem in the area, a special telephone service center for residents' complaints is available. Air sampling at the place of complaints and chemical analysis, along with measurement of meteorological parameters are also performed. According to the reports by the IP authorities to the Health Ministry Regional Office, a link between the odor and the IP emissions has been proved for communities located within 20 km. Based on this fact, we decided to consider the distance category "up to 20 km from the IP" as "exposed."

The Bedouin tribal settlements located on both sides of the 20 km boundary were defined as a stand-alone group. Thus the distance was categorized as follows: for the Jewish and Bedouin population: up to 20 km (proximal) and above 20 km (distant). Bedouin traditional tribal settlements located on both sides of the 20 km boundary were defined as intermediate.

An *additional exposure indicator *investigated in this study was the location of the community in relation to the dominant wind direction (north-west) from the IP. In this direction, we identified one proximal and two distant urban localities; two proximal and one distant small agricultural locality; one distant Bedouin permanent locality; two intermediate and one distant tribal amassment. These localities were compared with the localities in other directions from the IP, in each relevant stratum by distance from the IP.

*Data *on deliveries (live birth and stillborn) and parturient addresses were received from the Obstetrics Division's computerized files at the Soroka University Medical Center for each year of the observation period. In cases of lack of exact address in the electronic file, the information was retrieved from the patient's medical record.

Overall incidence of MCM and incidence for three sub-categories (MCM of the central nervous system, associated with chromosomal anomalies, and other MCM) were retrieved from the same data set and also from the files of the Neonatal Intensive Care Unit.

Since consanguineous marriages are very common in the Bedouin population, diagnoses of autosomal recessive diseases (ARD) were retrieved from the infants' medical records for 1999–2000.

### Statistical analysis

Calculation of MCM rate (with 95% confidence interval) was done by dividing the number of newborns born with MCM by the number of deliveries during the study period, according to place of residence. A comparison of the specific rates by population subgroup and by distance from the IP was done using the χ^2 ^test (or the Fisher exact test), relative risk (RR) with 95% confidence interval, and "test for trend." Significance of differences between groups was defined at less than 0.05. The study was approved by the local ethics committee, according to the Helsinki declaration.

## Results

### MCM in the Beer-Sheva subdistrict population, by distance from the IP and ethnicity

In all subdistrict localities located up to 20 km from the IP, 30,192 deliveries were recorded during the study period. In all subdistrict localities beyond 20 km from the IP, 31,853 newborns were recorded. The rate of MCM diagnosed at delivery in both groups was 4.6% (Table 1). The comparison of MCM rate by ethnicity (Bedouins vs. Jews) were significantly different (5.0% and 4.1% respectively, RR = 1.22, p < 0.001). Stratification by distance from the IP shows significant differences only in the Bedouin residents: 5.6% MCM rate in the proximal localities *vs. *4.8% in the distant ones (RR = 1.17, p = 0.006). The MCM rates in the Jewish population were similar at both distances from the IP, 4.1% and 4.2% respectively).

**Table 1:** MCM rates by ethnicity and distance from the IP in the Beer-Sheva subdistrict (1995–2000)

**Ethnicity**	**Distance from the IP**	**Number of deliveries**	**Number of MCM**	**MCMrate, (%)**	**RR (95%CI)**
Total population of the Beer-Sheva subdistrict^1^	Up to 20 km	30192	1376	4.6	1.00 (0.93–1.08)
	More than 20 km	31853	1451	4.6	
Total Bedouin population^2^		28524	1423	5.0	1.22*(1.10–1.30)
Total Jewish population		35326	1458	4.1	
Bedouin population	Up to 20 km^1^	9567	538	5.6	1.17**(1.04–1.29)
	More than 20 km	17152	831	4.8	
Total Jewish population	Up to 20 km	20625	838	4.1	0.96 (0.87–1.07)
	More than 20 km	14701	620	4.2	

^1 ^Not including residents on both sides of the 20 km boundary from the IP

^2 ^Including residents on both sides of the 20 km boundary from the IP

* p value < 0.001, ** p value < 0.01

### MCM among the Bedouin population, by distance from the IP and type of locality

The comparison of MCM rate for the period 1995–2000, by distance from the IP, ethnicity, and type of locality is presented in Table 2. The rates of MCM for the proximal and the distant traditional tribal settlements were 4.1% and 2.9% correspondingly. The difference was significant, and RR was equal 1.43 (95% CI: 1.10–1.85) for the first subgroup. For the intermediate traditional tribal settlements, MCM was recorded in 3.0% of the newborns. Compared to this rate, the rate for the proximal traditional tribal settlements was also higher (RR = 1.38, p = 0.03). Test of trend showed a high significance level (χ^2 ^= 8.95, p = 0.003) by proximity to the IP. In the proximal permanent localities the rate of MCM was 8.2%. The rate of MCM in the distant localities was 5.2%, which is significantly lower (RR = 1.63, 95% CI: 1.39–1.80).

**Table 2:** MCM rates by ethnicity, locality type, and distance from the IP in the Beer-Sheva subdistrict (1995–2000)

**Ethnicity and locality type**	**Distance from the IP**	**Number of deliveries**	**Number of MCM**	**MCM rate (%), (95% CI)**	**RR (95% CI)**
Bedouin traditional tribal settlements	Up to 20 km	6011	248	4.1 (3.6, 4.6)	1.43** (1.10–1.85)
	More than 20 km	2418	70	2.9 (2.2, 3.6)	
	Up to 20 km	6011	248	4.1 (3.6, 4.6)	1.38* (1.03–1.84)
	Both sides of the 20 km radius	1805	54	3.0 (2.2, 3.8)	
Bedouin permanent localities	Up to 20 km	3556	290	8.2 (7.3, 9.1)	1.63 (1.39–1.80)
	More than 20 km	14734	761	5.2 (4.8, 5.5)	
Jewish satellite localities	Up to 20 km	310	24	7.7 (4.8, 10.7)	1.79 (0.96–3.35)
	More than 20 km	347	15	4.3 (2.2, 6.5)	
Jewish urban localities	Up to 20 km	19899	799	4.0 (3.7, 4.3)	0.96 (0.85–1.07)
	More than 20 km	11928	499	4.2 (3.8, 4.5)	
Jewish agricultural localities	Up to 20 km	416	15	3.6 (1.8, 5.4)	0.83 (0.49–1.40)
	More than 20 km	2426	106	4.4 (3.6, 5.2)	

* p value < 0.05, ** p value < 0.01, p value < 0.001

### MCM among the Jewish population, by distance from the IP and locality type

The majority of the subdistrict deliveries occurred in the urban localities (31,827). The rates of MCM in the proximal and the distant subgroups were 4.0% and 4.2%, respectively (RR = 0.95, p = 0.48). Among the newborns of the single proximal Jewish satellite locality, MCM rate was 7.7%. In two distant similar comparison localities, the observed rate was 4.3%. The difference did not reach statistical significance (RR = 1.79, p = 0.09). The rate of MCM in the newborns from the Jewish proximal agricultural localities was not significantly lower than the rate calculated for the distant areas: 3.6% and 4.4%, respectively, (RR = 0.82) (see Table 2).

### MCM among the Beer-Sheva subdistrict population by ethnicity, distance, and dominant wind direction from the IP

The most frequent direction of the wind in the Negev area is north-west. Only a small number of localities are situated in this direction from the IP. No proximal or intermediate permanent Bedouin localities or traditional tribal settlements are sited in this direction. No significant differences in the incidence of MCM rates were found between subgroups placed in the direction of the predominant wind blowing from the IP and subgroups of comparison (data not shown).

### MCM in the Beer-Sheva subdistrict population by group of diagnoses, distance from the IP, and type of locality

Data describing the results of this chapter are presented in Table 3. Significantly increased risk of MCM of the central nervous system was found in the proximal Bedouin permanent localities, compared to the ones distant from the IP (RR = 2.27, 95% CI:1.44–3.60). The rates of MCM related to chromosomal anomalies among newborns from the proximal and intermediate Bedouin traditional tribal settlements were similar, 0.2%. No cases of these anomalies were recorded in newborns from the distant traditional tribal settlements. The differences for both comparisons were statistically significant. The rate of other, unclassified MCM in the proximal Bedouin traditional tribal settlements was significantly increased in comparison with the respective rates in the intermediate and distant groups: RR = 1.36 (1.04–1.79) and RR = 1.50 (1.09–2.07) correspondingly.

**Table 3:** MCM rates by groups of diagnoses, ethnicity, locality type and distance from the IP in the Beer-Sheva sub district over 1995–2000

**Groups of diagnoses**			**MCM of the central nervous system**				**Chromosomal anomalies**		**Other MCM**		
**Ethnicity & locality type**	**Distance From IP**	**Deliveries**	**Number of MCM**	**Rate (%)**	**RR (95%CI)**	**Number of MCM**	**Rate (%)**	**RR (95%CI)**	**Number of MCM**	**Rate (%)**	**RR (95%CI)**

**All Bedouin localities**	≤ 20 km	9567	43	0.45	1.38 (0.93–2.05)	19	0.20	1.42 (0.78–2.59)	419	4.37	0.92 (0.82–1.03)
	> 20 km	17152	56	0.33		24	0.14		808	4.71	
**Bedouin traditional tribal settlements**	≤ 20 km	6011	15	0.25	1.21 (0.44–3.32)	13	0.22*		220	3.66*	1.36 (1.04–1.79)
	> 20 km	2418	5	0.21		0	0.00		65	2.68	
	≤ 20 km	6011	15	0.25	0.75 (0.29–1.93)	13	0.22	0.98 (0.32–2.99)	220	3.66*	1.50 (1.09–2.07)
	± 20 km	1805	6	0.33		4	0.22		44	2.44	
	± 20 km	1805	6	0.33	1.61 (0.49–5.26)	4	0.22*		44	2.44	0.91 (0.62–1.32)
	> 20 km	2418	5	0.21		0	0.00		65	2.68	
**Bedouin permanent localities**	< 20 km	3556	28	0.99*	2.27 (1.44–3.60)	6	0.20	1.03 (0.42–2.53)	199	5.13	1.11 (0.95–129)
	> 20 km	14734	51	0.35		24	0.16		743	5.04	
**Jewish satellite localities**	≤ 20 km	310	0	0.00		1	0.32		23	7.42	1.72 (0.91–3.23)
	> 20 km	347	0	0.00		0	0.00		15	4.32	
**Jewish urban localities**	≤ 20 km	19899	22	0.11	0.60 (0.33–1.08)	15	0.08	0.56 (0.28–1.14)	762	3.83	0.99 (0.88–1.11)
	> 20 km	11928	22	0.18		16	0.13		461	3.86	
**Jewish agricultural localities**	≤ 20 km	416	0	0.00		0	0.00		15	3.61	0.87 (0.51–1.49)
	> 20 km	2426	4	0.17		2	0.08		100	4.12	
**All Jewish localities**	≤ 20 km	20625	22	0.11	0.60 (0.34–1.06)	16	0.08	0.63 (0.32–1.24)	800	3.88	0.99 (0.89–1.10)
	>20 km	14701	26	0.18		8	0.12		576	3.92	

* p value < 0.05, ** p value < 0.01, ± 20 km on both sides of the 20 km boundary

A comparison of ARD rates by distance from the IP and locality type in the Bedouin population are given in Table 4. A significantly lower rate of ARD was observed in the Bedouin infants from proximal traditional tribal settlements as compared to the infants in distant ones.

**Table 4:** ARD rates by locality type and distance from the IP in the Bedouin population (1999–2000)

**Distance from the IP**	**Number of deliveries**	**Number of cases**	**Rate per 1000 deliveries**	**95% confidence interval**
**Bedouin traditional tribal settlements**				
< 20 km	1409	3	2.13*	(-)0.28-4.54
> 20 km	604	10	16.56	6.38-26.73
± 20 km	474	5	10.55**	1.35-19.80
**Bedouin permanent localities**				
< 20 km	1635	4	2.44	0.05-4.84
> 20 km	5159	24	4.65	2.80-6.51

* p value < 0.05, ** p value < 0.01

± 20 km – on both sides of the 20 km boundary

### MCM among stillborn in the Beer-Sheva subdistrict population

The study included 511 stillbirths: 466 of them were dead before delivery (APD) and 45 died during delivery (IPD). The rate of MCM among the APD group was 20% while in the IPD group it was 31.1%. The overall rate of MCM among stillbirths in this study was 20.9%. No significant difference was detected in the rate of MCM among stillbirths according to distance from the IP (data not shown).

## Discussion

The average rate of MCM that we observed during the study period exceeded the range of 2–4% given by Dolk and Vrijheid, [19] summarizing data reported by Registries of Congenital Malformations. Among the 520,587 children who were born alive to New York state residents in 1996 and 1997, MCM represent 4.1% of live births, with a higher rate in black infants [20].

The findings of this study indicate a significantly higher rate of MCM among the Bedouin population, compared to the Jewish population of the Beer-Sheva subdistrict. Such preponderance is commonly associated with traditional consanguineous marriages [18].

In addition, the Bedouin mothers, being more traditional, are more reluctant to utilize ultrasound services and end a pregnancy even in the face of a prenatal diagnosis of MCM. We examined the rate of pregnancy terminations in Soroka Medical Center for the years 1996–2000. Of all pregnancy interruptions done during those years, 87.2% were performed to Jewish patients and 12.8% to Bedouins. During the same period 50% of the newborns in the region were of Arab-Bedouin origin.

Concerning the study hypothesis, statistically significant increased rates of MCM were observed among newborn Bedouins from localities closer to the IP compared with those that are more remote. The difference in MCM by distance is unlikely to be explained by difference in utilization of health care services since they are accessible every one. Moreover, the study design preserved the socio-economic level and residence type of the compared study groups.

In the absence of proximal Bedouin localities sited in the predominant wind direction from the IP, the role of this factor could not be assessed.

In epidemiological studies, American and West European literature provides some evidence on the increased risk of birth related to waste site [21-25]. Data on the association between the increased risk of birth defects and potential exposure to emissions from specific industries and various industrial facilities have been also reported [3-9,26,27]. In all the above-mentioned investigations, an indirect indicator of exposure (i.e., distance from a contaminating site or type of industrial activity) was used.

Ritz *et al. *[28] found that Odds Ratios for some cardiovascular malformations increased in a dose-response fashion with increasing first trimester exposure to CO or ozone. CO exists in emissions from the IP of the Beer-Sheva subdistrict.

In a single multi-site study (landfills, chemical dump sites, industrial sites, and hazardous treatment and storage facilities) in the San Francisco Bay Area, a 1.5-fold increase in risk for heart and circulatory malformations was detected in areas classified as having potential hazardous exposure [29].

It is necessary to take into consideration that the waste site in the IP serves as a pool for the entire country's industrial chemical hazardous material. The combination of this landfill site with the 17 plants at the IP may create more complicated ecological pressure and enhance the possibility for an extended area of diffusion, especially under conditions of temperature inversions, which are common in our subdistrict.

The association between increased MCM rates and residential proximity to the IP only for the Bedouin population could be explained by their increased vulnerability due to low socio-economic level, dwelling in tents, or in other poor housing facilities in the traditional tribal settlements, and being outdoors for their daily activities during most of the day. Bedouins and especially Bedouin women are much more confined to the place of residence than Jewish women, as most of Bedouin women are housewives.

Moreover, a traditional open fire is used for cooking and heating and paternal cigarette and hookah smoking might increase the burden inflicted by inhalation of the chemical mixture and increase the combined toxicity.

One of the explanations may be, as mentioned above, the reluctancy of Bedouin women to utilize ultrasound and to terminate pregnancies, even in cases of MCM diagnosis by ultrasound.

### Methodological issues inherent to the study

Ecologic bias defined as the "failure of ecologic effect estimates (difference in outcome rates across groups) to reflect the biological effect at the individual level" [30], does not allow inference of causality.

One source of this ecologic bias is within-group bias that may occur due to misclassification. Exposure misclassification related to the use of distance as an indirect indicator of exposure cannot be excluded. As for the Bedouin population, for which we found an increase of MCM rates with proximity to IP, underestimation could be suggested as we could not measure the risk for a continuous distance. Exposure misclassification due to residential mobility during pregnancy may be related to moving to permanent localities. But, as a rule, this process takes place within the same zone classified by distance from the IP. Thus, regarding the Bedouin tribes residing out of permanent localities, underestimation may also be considered. Misinformed address (about 10%) should be related to non-systematic bias.

We tried to avoid extraneous risk factors such as ethnicity and socio-economic status by the relevant stratification of the population studied: a) the Arab-Bedouin and the Jewish populations; b) the permanent localities and the tribal traditional settlements for the Bedouin population, urban, agricultural, and satellite localities for Jewish. We assumed that this design should reduce cross-level (between-group) bias attributed to confounding and effect modification by group,

An additional cross-level bias could be associated with the contextual effect of the exposure variable of living near the IP versus being exposed to its emissions. This effect might differ from the biological effect of the individual-level analogue [30]. We can speculate that since Bedouins, and especially the women, are more confined to their place of residence, this bias should be ascribed to a lesser extent to the Bedouin population than to the Jewish. This might serve as an additional explanation for the lack of difference in MCM rate according to distance from the IP among urban Jewish newborns, but not for Jewish agricultural localities.

The factors which are well-known and widely accepted to be associated with birth defects could be considered as possible confounders in our study: maternal age, health status, infections, diet or stress during pregnancy, paternal smoking habits, use of medication, illicit drugs, alcohol, and pesticides (occupationally or at home), other types of occupational and household exposure.

Regarding the avoidance of ascertainment (outcome) bias, it is important to indicate that data on deliveries and MCM were registered routinely and meticulously in the single regional hospital. Health care services are available and accessible for the entire population.

It should be taken into consideration that the MCM reported in this study were usually detected during the prenatal period by ultrasound. Without prenatal ultrasound detection of MCM, newborns might be regarded as healthy, so that reduced use of this service might reduce the reported number of MCM at birth, especially in the Bedouin newborns.

This study did not include information regarding pregnancies that were terminated before the 22nd week. The Soroka University Medical Center defines a delivery as a termination of pregnancy after the 22nd week and/or the birth of a newborn weighing 500 gr or more. Prenatal diagnosis of MCM will often bring about the premature termination of the pregnancy, usually before it is regarded as a delivery and added to the maternity department database, resulting in a reduced overall incidence of MCM.

According to the modern paradigm, congenital malformations are caused by interaction of genetic and environmental factors [31]. Genetic susceptibility among the Bedouin population may be, first of all, related to consanguineous marriages. In a survey of 649 Bedouin couples conducted in 1991–92, 36% of the marriages were between first cousins and a total of 60% of the marriages were considered consanguineous [32].

Our results show that the autosomal recessive disease (ARD) rate is significantly lower in the Bedouin newborns from localities proximal to the IP. This finding indicates that the inhabitants in close proximity to the IP do not have higher rates of consanguineous marriages, which could be considered as an important confounder. As for the lower ARD rates among the Bedouins residing close to the IP, we can assume that the damaged genetic makeup makes fetuses more vulnerable to the IP exposure and thus increases the rate of the aborted fetuses with ARD. In addition, due to the small numbers of observations in the newborns with ARD, the statistical power of the findings is relatively low. The same explanations might be considered in evaluating the absence of difference in the observed rates of MCM among the stillbirths by distance from the IP.

The *statistical power of the results *is low for diagnosis of subgroups of MCM, thus, our conclusion is related to findings on MCM as a group.

## Conclusion

The results of this study suggest an association between residential proximity to the regional IP and increased risk for MCM in the Bedouin newborn population. These data provide basic figures suggesting a need for the initiation of preventive measures and active reproductive outcomes monitoring systems.

The findings, as well as public concern about the adverse health effect of exposure to the regional IP, justify further investigations to evaluate the role of regional industrial pollution in MCM rate increase, and to enforce strict regulations to reduce and prevent industrial park emissions.

## Abbreviations

1. ARD – Autosomal recessive disease

2. ATSDR – Agency for Toxic Substances and Disease

3. IP – Industrial park

4. MCM – Major congenital malformations

5. RR – Relative risk

## Competing interests

The author(s) declare that they have no competing interests.

## Authors' contributions

YB carried out data acquisition, contributed to study design and manuscript drafting.

EK contributed to study conception, design, and coordination, data analysis and interpretation, and manuscript drafting. RH participated in data acquisition and manuscript drafting. IB participated in results interpretation, manuscript revising for important intellectual content. MP and NB contributed to data acquisition and analysis. BS was the principal investigator of the project. She contributed to study conception and design, data interpretation, manuscript drafting.
